# Effects of first aid training in the kindergarten - a pilot study

**DOI:** 10.1186/1757-7241-19-13

**Published:** 2011-02-28

**Authors:** Georg Bollig, Anne G Myklebust, Kristin Østringen

**Affiliations:** 1 Department of Surgical Sciences, Haukeland University Hospital, University of Bergen, Bergen, Norway; 2 Bergen Red Cross Nursing Home, 5043 Bergen, Norway; 3 Hellemyren kindergarten, 5043 Bergen, Norway

## Abstract

**Objective:**

Children can be the only persons present in an emergency situation. Aim of the study was to evaluate the effects of a first aid course for 4-5-year-old kindergarten children given by a first aid instructor and kindergarten teachers.

**Methods:**

A mixed methods approach using both quantitative and qualitative methods was used to investigate the effects of teaching first aid in the kindergarten in the present study. 10 kindergarten children at the age of 4-5 years were included in a pilot-study, 5 girls and 5 boys. Three of them were four years and seven were five years old. Two months after completion of the first aid course children were tested in a scenario where the children had to provide first aid to an unconscious victim after a cycle accident. The next seven months the children were followed by participant observation.

**Results:**

The findings suggest that 4-5-year-old children are able to learn and apply basic first aid. Tested two months after course completion 70% of the children assessed consciousness correctly and knew the correct emergency telephone number; 60% showed correct assessment of breathing and 40% of the participants accomplished the other tasks (giving correct emergency call information, knowledge of correct recovery position, correct airway management) correctly. Many of the children showed their capabilities to do so in a first aid scenario although some participants showed fear of failure in the test scenario. In an informal group testing most of these children could perform first aid measures, too. Teaching first aid also lead to more active helping behaviour and increased empathy in the children.

**Conclusion:**

Kindergarten children aged 4-5 years can learn basic fist aid. First aid training should start in the kindergarten.

## Introduction and background

Laypersons are an important factor for saving lives in emergency situations. According to Eisenburger and Safar Life-Supporting First-Aid (LSFA) should be part of basic health education and all persons from the age of 10 should learn LSFA-skills including Basic Life-Support (BLS) and cardiopulmonary resuscitation (CPR) [1]. One important barrier and main concern of laypersons about giving first aid to acute ill or injured people is the fear to make mistakes. In Austria 68% of the participants of a study (n = 597) stated that they would not provide first aid because they feared to do something wrong [2]. Several studies have shown a clear relationship between the level of first aid training and the quality of first aid measures provided [2-4]. This underlines the importance of first aid training for the public.

Unfortunately first aid training does not increase the rate of helping [4]. Therefore the motivation to help others is paramount and the helping rate can probably be increased by first aid courses that include strategies to overcome inhibitors of emergency helping behaviour [4].

There are many examples of children who have provided first aid measures or saved lives by recognizing life-threatening emergency situations in the media. In a number of cases small children have saved the life of a parent at home just by giving an emergency call and informing the Emergency Medical Service (EMS) or the Fire Department. In a recent case from Germany a four-year-old girl saved the life of her 31-year-old mother, who suffered from hypoglycaemia by calling for help at night-time [5]. This case illustrates that a young child can be the only person present in case of an emergency and that first aid education therefore should start as early as feasible. Several authors have documented that school children can learn and provide first aid and life supporting first aid measures and have advocated that primary school children should learn first aid in school [6-9]. An own study of primary school children demonstrated that 6-7-year-old children can give basic first aid to an unconscious patient and that a first aid course with 5 lessons leads to a significant increase in both - first aid knowledge and skills [8]. This course included airway management and application of the recovery position. The conclusion from this study was that primary school children should receive first aid training starting in the first grade [8].

Aim of the present study was to evaluate the effects of a first aid course for 4-5-year-old kindergarten children given by a first aid instructor and kindergarten teachers.

## Methods

A mixed methods approach using both quantitative and qualitative methods was used to investigate the effect of teaching first aid in the kindergarten in the present study [10]. The mixed methods approach combined quantitative data from testing the participants in a test scenario and qualitative data derived from field notes taken in the kindergarten. They were taken during the course and the following seven months after the course to investigate the effects of first aid training on the children's behaviour in everyday life. The field notes were written and collected by two kindergarten teachers who both actively participated in teaching first aid to the children in the study group. The methods used for the qualitative part of the study and data analysis were "qualitative description" and "qualitative content analysis" [11]. The main reason for using mixed methods in this pilot study was to provide a bigger and richer picture of the effects first aid teaching has on the children in the kindergarten. Field notes help to show which effects the training had besides the effects on practical skills and knowledge, which were quantitatively tested in a first aid scenario. Another reason was to compare the findings from the quantitative and the qualitative approaches.

**Figure 1:**
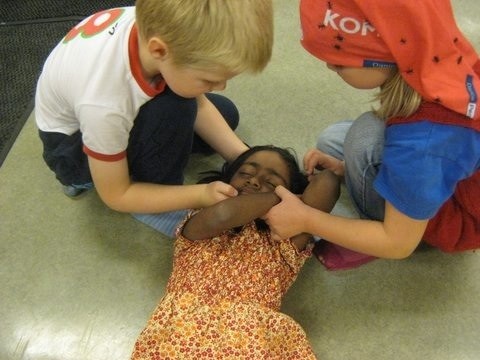
**Children performing first aid**.

The study group received a first aid teaching program consisting of 6 lessons (30-40 minutes each). The course was lead by the first author who is first aid instructor, paramedic and anaesthesiologist with more than 25 years experience in teaching first aid and more than 15 years in teaching first aid to children. In every lesson one kindergarten teacher worked as assistant instructor. A glove puppet was used to ease the contact to the children. A new lesson was performed once a week. The teaching program was similar to that used in a previous study on primary school children aged 6-7 years [8]. It was adapted to the needs and abilities of 4-5-year-old children to introduce elementary knowledge of first aid. Figure 1 shows children practicing first aid during the course.

The main difference between the present course and the course used before [8] was that the present course consisted of 6 lessons instead of 5 and that the duration of the lessons was shorter (30-40 min). This was changed due to the different capacity to pay attention for a longer time. The curriculum of the teaching program included basic first aid knowledge and was almost the same as in a previous study on primary school children [8]. The course curriculum is shown in additional file 1. Cardiopulmonary resuscitation including mouth-to-mouth/mouth-to-nose breathing, chest compressions and defibrillation were not part of the course. An important part of the teaching was learning the "five-finger-rule" to basic first aid (additional file 2).

Testing was based on the same scenario as used in our previous study [8], where the children had to assist an unconscious child involved in a bicycle accident without any help from others. The instructor told the tested children "A friend of yours has fallen from the bicycle and hurt his head. He is lying still on the ground and does not move. What are you going to do?" Questions from the children were not answered and no other help was given in order to accomplish the first aid measures. The children had to decide and to act on their own. One child played an unconscious victim. Children from the study group were tested two months after course participation. The children's performances in a first aid scenario were registered as tasks accomplished or not. Tested items are shown in table 1.

**Table 1:** Tested items in the first aid scenario

**Task nr**.	First aid measure	Criteria for correct task
1	Correct assessment of consciousness	The child had to talk to the victim and to try to wake him up

2	Correct assessment of breathing	The child had to look, listen and feel the breath

3	Knowledge of the correct emergency telephone number	The child had to tell the correct emergency telephone number (which is 113 in Norway)

4	Giving correct information for the emergency call	The child had to tell the dispatcher what had happened and had to give the correct location of the accident

5	Performance of correct recovery position	Performance of correct recovery position

6	Correct airway management with open airway	The child had to tilt the head backwards

The study was performed in the kindergarten "Hellemyren barnehage" in Bergen, Norway. In this kindergarten there are 22 children divided into two groups according to their age. All children aged 4-5 years from this kindergarten were included in the study group after their parents gave written informed consent.

### Ethical considerations

As the project was an evaluation of a teaching course it was out of the mandate of the research ethics committees in Norway and did not need approval from a research ethics committee according to Norwegian law and regulations. The participating children, their parents and teachers did get written and oral information before the start of the study. They were informed about the right to quit at any time without the need for an explanation and without any consequences for them. The children's parents gave written informed consent before entering the study.

## Results

10 children were included in the study, 5 girls and 5 boys. Three of them were four years and seven were five years old. Results of the test performed two months after course completion are shown in table 2 compared to results from other studies [2,7,8].

**Table 2:** Test results - percentage of children age 4-5 who fulfilled the given tasks 1-6 correctly (n = 10) compared to results from the literature

	Results from the present study		Results from the literature		
		
Task no	Number of children who fulfilled the task correctly from the present pilot study: children age 4-5 (n = 10)	Success rate in % from the present pilot study: children age 4-5 (n = 10) tested 2 months after course completion	**Success rate in % from a study using a wall calendar as teaching aid in primary school: children age 5-6 (n = 226) (ref. **[7]**)**	**Success rate in % from own previous study: children age 6-7 (n = 117) tested directly after course completion (ref. **[8]**)**	**Success rate in % from a study using adults (n = 182) (ref. **[2]**)**
		
1. Correct assessment of consciousness	7	70%	17%	49%	83%
**2. Correct assessment of breathing**	6	60%	Not tested in this study	79%	46%

**3. Knowledge of the correct emergency telephone number**	7	70%	To inform an adult or to dial 113 on a telephone was	77%	87,5%
			considered as success. This was accomplished by 79%.		
**4. Giving correct information for the emergency call**	4	40%		50%	74%

**5. Performance of correct recovery position**	4	40%	64%	87%	Not tested in this study

**6. Correct airway management with open airway**	4	40%	31%	68%	30%

70% of the children assessed consciousness correctly and knew the correct emergency telephone number. 60% were found for correct assessment of breathing and the other tasks were correctly accomplished of at least 40% of participants.

In the test scenario many children showed fear of failure although they were familiar with the testing persons, who were the same as involved in teaching (all authors). In contrary to the test results the field notes and observations in everyday life showed that also some of the children who did not show their capability to provide first aid in the scenario did know what to do. They were able to provide first aid measures when tested informal in a group play situation instead of the formal first aid scenario where they were alone in a room with the investigators and one child who played the victim.

The field notes taken during and after the course showed that first aid became an important topic for the children and was spontaneously included in playing activities. This included that the course participants taught first aid to the other children in the kindergarten. Some examples from the field notes will be presented:

### Working together

**Figure 2:**
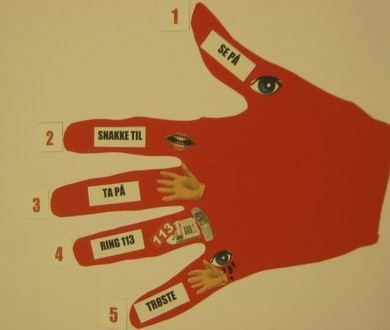
**The five-finger-rule poster**.

As aid to remember the "five-finger-rule" to basic first aid a poster was designed together with the participating children. Because of the fact that the children were not able to read written language, a poster of a hand with pictures for the five items was developed. The pictures were based on the children's suggestions and the agreement to it from the group. This led to a poster mnemonic, which consists of both - written language and pictures. The content of this poster was reassessed in a discussion with the children seven months after the course and the children concluded to adapt the poster. Changes were that point four included two different types of telephones and that point five was expanded and should include both - an eye with tears and a hand, which illustrates to help and to comfort when somebody is crying (additional file 3 and figure 2).

"As a kindergarten teacher pretended to be unconscious in the playing yard without giving prior information to the children around, they used a team approach to help her. They checked her breathing and laid her in the recovery position working as a team without an adult present. They tilted the had backwards and discussed in the team that the reason for that was that vomit could come out instead of blocking the throat."

### Role of first aid in everyday life

The poster presenting the "five-finger-rule" (figure 2) has been hung up on the wall in the kindergarten and is frequently used to refresh first aid knowledge (comparable to the posters of the European Resuscitation Council).

First aid has become an everyday issue in the kindergarten and repetition of first aid knowledge is done on a regular basis using the poster, which was developed during the course with the help of the participating children.

Some of the three-year-old children in the kindergarten who had witnessed parts of first aid training through a window started to include first aid scenarios in their everyday play. By imitating the older children they also learned some basic first aid measures.

"One three-year-old observed the older children practising first aid and the recovery position. So she lay down and played an unconscious person without being asked to do so. Another three-year-old came to help her. The kindergarten teacher asked what one should do and the three-year-old said that one should check if she was breathing and that she should be laid in "this position" (searching for the word recovery position). Asked about the importance of tilting the head backwards the child said that vomit might run out of the mouth in this position."

"When a famous Norwegian artist died the children spontaneously asked if someone did supply first aid to him, who had put him into the recovery position, who had informed the Emergency Medical Service and who had comforted him before he died."

This illustrates the fact that children have a natural proficiency for empathy. Although somebody had already died they were interested in how people tried to help and to comfort him when dying.

### Teaching first aid to others and showing their skills

The children who attended the course were proud to have learned first aid and to be able to save lives. Some taught their knowledge and skills to others like other children or members of their family.

"One girl told her grandfather at home that he should lay himself on the ground. He did not know about the first training in the kindergarten. Then she checked his breathing and laid him in the recovery position. After that she told him about the importance of this first aid measure"

## Discussion

The main finding of this study is that 4-5-year-old children are able to learn and apply basic first aid. Many of the children showed their capabilities to do so in a first aid scenario. Although the participants were familiar with the persons testing them, many were reluctant to perform first aid in the scenario, whereas they showed both - first aid knowledge and skills when observed in play situations in everyday life. Therefore starting first aid training in the kindergarten seems worth the effort and can probably lead to a more positive attitude towards giving first aid and increase the rate of helping. These assumptions have of course to be investigated and proved in future studies.

Different researchers have documented that first aid training of school children leads to increased knowledge and first aid skills [6-8]. Several authors have recommended that first aid training should start early in life and that primary school children can learn to provide first aid [6,8,9,12-14]. Negative consequences of first aid training as e.g. anxiety among the children, contact with Emergency Medical Services without being in a real emergency, etc. are concerns that opponents to first aid education for children will state. Although one should think about these issues, negative consequences of first aid education for children have not been reported to our knowledge.

Our results showed that many children had fear of failure in the test situation and this can certainly have influenced our results. The test situation was somehow unknown to the children and the testing one by one might have led to a raised stress level. When the children were tested again using a first aid scenario in an everyday play situation interestingly most of them knew what to do and were able to perform first aid measures as e.g. the recovery position. Taken into account the children's reaction the results might be even better without this reaction. This supports our conclusion that children in the kindergarten can learn and perform basic life-saving first aid measures. One possible explanation for this behaviour in the test situation could be the development of fear of failure in test situations, which were experienced as test. From group psychology it is known that children behave differently if they are together with other children or adults instead of being alone and that status in the group plays an important role for their expectations [15]. This is of importance too, for the expectations one has to oneself and for one's performance [15]. One explanation might be that the children's own expectations were influenced by the absence of the other group members and that this caused a stress reaction leading to a poorer performance than shown in the group. It is unclear whether this possible reaction to the test situation would occur in a real emergency situation. It would be interesting to do a follow-up-study in order to investigate whether the children have used their first aid knowledge in real life. The test scenario used in this study can only measure practical skills. It is unclear whether the skills and knowledge displayed in this test lead to an enhanced ability and motivation to provide first aid in a real emergency situation.

A Swedish study using questionnaires including 2800 randomly selected people has shown that 30% of respondents had used their first aid skills in practice after initial first aid training [16]. Although experts and instructors on first aid will agree that doing nothing is more dangerous than doing something which might be incorrect many people are afraid of providing first aid because they fear to do something wrong [2]. Learning first aid should therefore include both knowledge-transfer and motivation to give first aid [9]. To start first aid education in the kindergarten could probably lead to first aid as a normal activity of daily life which everybody will apply if motivated. Our results suggest that learning first aid in the kindergarten leads to including this topic as everyday life activity. The qualitative descriptions of everyday situations showed the children's natural proficiency for empathy. It has been stated that empathy is one of the most important qualities which children have from birth on and it must be developed in childhood, adolescence and further throughout life. The surrounding adults' behaviour can either strengthen or weaken the development of an empathic attitude in the children [17]. In our study group we observed a more active behaviour of the children to help and to comfort others in daily life. We think that teaching first aid has led to positive changes in social responsibility and empathic behaviour in the children in addition to acquiring first aid knowledge and skills.

In our previous study we could show that skill retention tested 6 months after the course was significantly better for five out of six tested tasks compared to children with no course [8]. There definitely is a need for repetition of first aid knowledge and skills [9,18]. A simple measure introduced in the Hellemyren kindergarten in Bergen is the first aid poster at the wall. This poster can help to remember in repetition sessions as well as in an acute situation. Used once a month it helps to raise awareness about the importance of giving first aid and needed algorithm of applying first aid. The poster serves the same purpose as first aid and CPR posters of the ERC displayed in hospitals or public places.

What we need in the future is a focus on first aid and motivation to apply first aid knowledge. Common European or international working groups could help to increase scientific research in this field and to establish consensus and guidelines for teaching first aid [19-21]. These efforts should include teaching first aid to kindergarten children.

### Limitations

The study covers only of a small group of children (n = 10) and therefore it might not be representative for the whole population of kindergarten children all over the world. The results were not compared to a control group. This was done because of several reasons. First we do not believe that a control group would have added more knowledge to our present pilot study that was based on a mixed methods approach. Secondly a control group without first aid training has been used before in a study on primary school children published in Resuscitation by Bollig et al. 2009. The present study was conducted by kindergarten employees and the instructor, who could likely have influenced the supervision offered in the follow-up period. To avoid this to bias the observations made by the research team theses topics were discussed in team reflections during the evaluation period. Nevertheless the mixed methods approach led to a richer picture than just testing first aid knowledge and skills. Despite these limitations the results from this pilot study are promising and further research on this topic seems appropriate and justifies the verification of our findings by using bigger study populations.

## Suggestions for further research

Further research projects should focus whether first aid training starting in kindergarten increases the helping rate in emergency situations. Longitudinal studies with follow up over many years could show whether first aid training early in the kindergarten and primary school changes the helping rate in real emergency situations.

It is unclear whether first aid should be taught by teachers who are not certified first aid instructors or by certified instructors. It would be interesting to investigate the effect of both approaches on the children's motivation to help in a real emergency situation.

## Conclusions

First aid training of 4-5-year-old children in the kindergarten is feasible and leads to increased knowledge, skills and most important motivation to provide first aid. Knowledge and skill retention tested in play situations in everyday life is good. It is suggested that first aid training should already start in the kindergarten.

## Abbreviations

LSFA: Life-Supporting First-Aid; BLS: Basic Life-Support; CPR: Cardiopulmonary Resuscitation; EMS: Emergency Medical Service; ERC: European Resuscitation Council;

## Competing interests

The authors declare that they have no competing interests.

## Consent

The children's parents for all children participating in the study gave written informed consent. In addition written informed consent for publication of this report and accompanying images was obtained. A copy of the written consent (in Norwegian) is available for review by the Editor-in-Chief of this journal.

## Authors' contributions

GB developed and adapted the curriculum. All authors contributed to designing, drafting and writing the manuscript. All authors read and approved the final manuscript.

## Supplementary Material

Additional file 1 **Course curriculum**. Click here for file

Additional file 2 **The "five-finger-rule" to basic first aid**. Click here for file

Additional file 3 **Description of pictures included in the revised five-finger-rule poster**. Click here for file
