# Supplementary material for: Effects of first aid training in the kindergarten - a pilot study
Source: Scand J Trauma Resusc Emerg Med. 2011 Feb 28;19:13. doi: 10.1186/1757-7241-19-13 (PMC3060136; doi:10.1186/1757-7241-19-13)
Supplement: Additional file 1 — Course curriculum. [file 1757-7241-19-13-S1.DOC]

**Course curriculum**

The lessons included the following topics:

1. lesson: the body and its functions; assessment of consciousness and breathing

2. lesson: wound treatment, bleeding

3. lesson: unconsciousness, open airway and recovery position

4. lesson: behaviour in emergency situations; emergency call

5. lesson: repetition and first aid scenarios

6. lesson: repetition and first aid scenarios

7. lesson: test of performance in a first aid scenario
