# Supplementary material for: Effects of first aid training in the kindergarten - a pilot study
Source: Scand J Trauma Resusc Emerg Med. 2011 Feb 28;19:13. doi: 10.1186/1757-7241-19-13 (PMC3060136; doi:10.1186/1757-7241-19-13)
Supplement: Additional file 2 — The "five-finger-rule" to basic first aid. [file 1757-7241-19-13-S2.DOC]

**The “five-finger-rule” to basic first aid**

1. Look at him
2. Talk to him
3. Touch him (in order to try to wake up a sleeping person)
4. Call 113 (the telephone number of the Norwegian Emergency Medical Service)
5. Give comfort and stay with him
