# Supplementary material for: Effects of first aid training in the kindergarten - a pilot study
Source: Scand J Trauma Resusc Emerg Med. 2011 Feb 28;19:13. doi: 10.1186/1757-7241-19-13 (PMC3060136; doi:10.1186/1757-7241-19-13)
Supplement: Additional file 3 — Description of pictures included in the revised five-finger-rule poster. [file 1757-7241-19-13-S3.DOC]

**Description of pictures included in the revised five-finger-rule poster**

1. A picture of an eye representing “look at him“
2. A picture of a mouth representing “talk to him“
3. A picture of a hand representing “touch him“
4. A picture of two different telephones representing “call 113“
5. A picture of an eye with tears and a hand representing “Give comfort“
